# Decoding the human phageome helps to unravel microbial dynamics in health and disease

**DOI:** 10.1128/aem.00919-25

**Published:** 2025-08-01

**Authors:** Martha J. Vives

**Affiliations:** 1 Department of Biological Sciences, Universidad de los Andes 117374, Bogotá, Colombia; Michigan State University, East Lansing, Michigan, USA

## Abstract

Bacteriophages are the most abundant biological entities on Earth—equally abundant within the human body. Their potential impacts on host function and homeostasis are becoming increasingly important in biomedical and microbiological research. Phage abundance and diversity often signal changes in the resident bacterial microbiome, but they can also regulate microbiome dysbiosis, help identify pathogenic bacterial candidates, and provide clues to understand the disease-related microbiome changes and the role of the phageomes in health and disease contexts. In a recent minireview, Rybicka and Kaźmierczak (Appl Environ Microbiol 91:e01788-24, 2025, https://doi.org/10.1128/aem.01788-24) synthesize the current understanding of phage communities across the human gut, oral cavity, skin, respiratory, and urogenital tracts, seeking connections with health and disease status.

## COMMENTARY

Bacteriophages or phages, viruses that exclusively infect bacteria, have provided essential research tools to advance on fundamental questions in biology: the nature of the molecule coding for hereditary traits (1), the principles of sequence-specific recognition and restriction by nucleases (2), the process of bacterial DNA replication (3), gene regulation and operons (4), and DNA sequencing (5), among other breakthroughs.

To understand how phages interact with their bacterial host cell, scientists have focused research efforts on the study of their life cycles. These studies led to the description of the life cycles on lytic and lysogenic, with T4 and Lambda phages providing representative models, respectively. But there are additional lifestyles to consider: chronic infection, carrier state, and pseudolysogeny (6), widening the spectrum of potential interactions between a virus and a bacterium. In chronic infection, viral particles are excreted continuously through the cell envelope without killing the host cell; the genome may integrate into the host genome or remain in the cytoplasm. Examples of chronic infection are from filamentous ssDNA phages. The carrier state has been described for RNA phages, defined as the persistent infection in which phage particles are produced and accumulated in the host cell cytoplasm without release. Pseudolysogeny remains somewhat enigmatic and the subject of scientific debate: the phage replication is arrested during or after the uptake of the phage genome; however, the infection process may be stopped at a later stage as well.

The classical view of phages as parasites has also been revised. Phages are now recognized as bacterial symbionts (7), a shift supported by a range of phenomena such as lysogenic conversion, a process where prophages inserted in the bacterial genome provide their hosts with new properties. New functionalities acquired via lysogenic conversion include the synthesis of toxins and other virulence factors; the transfer of genetic material among bacteria through transduction; protection from other viruses by superinfection exclusion; biofilm induction; and elimination of competitors and release of nutrients, among other acquired functions. The expanded view of phage-host interactions, together with the extraordinary abundance, ubiquity, and ecological impact of phages, highlights the need for deeper investigation into the diversity of interactions modulating phage–bacterium dynamics.

Progress in understanding phage ecology has been hindered by several challenges, notably the vast diversity of uncultured microbial hosts, the absence of a viral analog to the SSU rRNA gene for phylogenetic profiling, and the polyphyletic origins of viruses. Developments in sequence-based methods, along with improvements in phage genome analysis and gene annotation, have significantly enhanced our capacity to predict and understand the importance of the viral component in Nature. Based on these advances, an international consortium under the International Committee on Taxonomy of Viruses (ICTV) recently introduced a 15-rank hierarchical framework of taxonomy that organizes viruses in seven distinct realms (Table 1), that acknowledges the lack of a universal ancestor. Phages are currently found in five of these realms: Duplodnaviria, Monodnaviria, Varidnaviria, Riboviria, and Singelaviria. For instance, the well-characterized phages T4, T7, and Lambda are placed within the families Straboviridae, Autotranscriptaviridae,  and class Caudoviricetes (with Lambda still awaiting formal family assignment), respectively; all belong to the realm Duplodnaviria.

**TABLE 1:** Viral realms and representative phages^*a*^

Realm	Genome	Characteristic gene/feature	Virion morphology	Examples of phage families (representative virus)
Duplodnaviria	dsDNA	HK97-fold major capsid protein	Binary	Class Caudoviricetes (Lambda phage), Straboviridae (T4 phage), Autotranscriptaviridae (T7 phage)
Varidnaviria	dsDNA	Double jelly-roll major capsid protein	Cubic	Tectiviridae (PED1), Corticoviridae (PM2)
Monodnaviria	ssDNA	Superfamily HUH endonuclease	Cubic, helical	Microviridae (φX174), Inoviridae (M13)
Riboviria	ssRNA or dsRNA	RNA-dependent RNA polymerase or reverse transcriptase	Cubic	Fiersviridae (MS2, Qβ), Cystoviridae (Φ6)
Adnaviria	dsDNA	Distinctive alpha-helical major capsid protein	Helical	No bacteriophages identified; archaeal viruses only
Ribozyviria	Circular ssRNA	Ribozyme-encoding; depends on helper virus	Uses the helper virus capsid	No bacteriophages identified; satellite virus (hepatitis D virus)
Singelaviria	dsDNA	Single jelly-roll major capsid protein	Cubic	Matsushitaviridae (*Thermus* phage IN93), archaeal viruses

^*a*^ This table was constructed based on information available in the ICTV New Taxonomy Release: MSL40 (8).

Historically, human microbiome studies have focused mainly on the bacterial diversity, abundance, as well as their dynamics, particularly their shifts under disease conditions compared to healthy states. However, bacteriophages are the most numerous viral components within the human body, influencing either directly or indirectly the numbers, activity, and interactions of the bacterial communities. From this recognition, studies on the phageome—a subcategory of virome that comprises the total community of phage populations—(9) have gained importance.

Human body niches harbor unique and specific microbial communities, phages included. But what roles do they have in our bodies? How do they impact human health? Do they play a role in diseases? In their recent minireview published in *Applied and Environmental Microbiology* (AEM) (10), Rybicka and Kaźmierczak synthesize the current understanding of phage communities across five human niches (gut, oral cavity, skin, respiratory, and urogenital tracts) looking for connections with the health and disease status. The authors are affiliated with the Hirszfeld Institute of the Polish Academy of Sciences, a cornerstone in both historic and modern biomedical research, immunology, and experimental therapy. Established by Ludwik Hirszfeld, the institute has continued the pioneering work on phages initiated by its founder to become a leader in phage therapy (11). In the AEM article, the authors review the complex dynamics between phages, bacteria, and the human host that can be harnessed as diagnostic and therapeutic tools.

Harnessing the human phageome in diagnostics and therapeutics of human disease is challenging. The human body harbors a staggering number of viruses (10^9^–10^10^ viral particles/g feces and 10^5^ virus-like particles/ul saliva) (10). Reports on differential viral patterns in colorectal cancer patients compared to healthy individuals suggest an indirect impact on cancer development by altering the resident bacterial community (12, 13). Building on this idea, researchers have proposed that modulating the mucosal virome could offer therapeutic avenues for inflammatory conditions such as Crohn’s disease, inflammatory bowel disease, and irritable bowel syndrome (14). This proposal has grounds on experiments showing that fecal viral transplantation restores gut balance (15). Another promising research direction involves the role of phages in the gut–brain axis. Studies suggest that fecal microbiota transplantation from humans enriched in phages within Caudovirales (now reclassified as Caudoviricetes) phages could improve memory performance in mice (16). Remarkably, supplementation with one specific Siphoviridae phage enhanced memory in *Drosophila* model (16), opening new possibilities for phage-based interventions beyond the gut.

Significant progress has been made in deciphering the human gut phageome. Dedicated resources, such as the Gut Virome Database, the Metagenomic Gut Virus catalog, and the Gut Phage Database, allow for systematic analyses and comparative studies of complex phage data sets at an unprecedented scale. Rybicka and Kaźmierczak draw attention to the scarcity of information about oral, skin, respiratory, and urogenital phageomes (10), and invite further exploration of these underexamined niches. There are interesting correlations connecting dental caries, gingivitis (17), and the presence of the pathogen *Gardnerella vaginalis* (18) with altered phageomes.

Phageomes are, indeed, “broad and alien worlds.” Bacteriophages are found across all environments: animals, plants, soils, rivers, lakes, and oceans. Furthermore, phages can significantly impact ecosystems productivity as well as economic activities. For example, marine cyanobacterial phages can carry genes coding for essential components of photosystem II, preventing photo-inhibition and allowing photosynthesis to proceed (19). In Lake Nakuru, Kenya, phages infecting and killing *Arthrospira fusiformis*, the main component of the lesser flamingo (*Phoeniconaias minor*) diet, have been linked to the dramatic declines in flamingo population (20). In industrial poultry production, phage cocktails such as SalmoFree cocktail demonstrated the ability to control *Salmonella* in the chicken gut. Intriguingly, reductions in *Campylobacter* abundance were also observed, despite the phages not infecting this bacterial genus, suggesting the possibility of indirect, metabolically mediated effects (21).

Further research is essential to answer questions regarding the mechanisms that connect phages and health. Current evidence demonstrates both direct and indirect interactions between phages and the vertebrate immune system (22). In one example from full-scale egg production farms, administration of phages targeting *Salmonella* serovar Gallinarum was correlated with an increase in egg production per bird (personal communication, S. Hernández, 2025). These systemic outcomes suggest that phages may influence broader aspects of animal homeostasis beyond *Salmonella* infection control.

In conclusion, phages are far from passive inhabitants of the human microbiome. Instead, they are active players with the potential of shaping microbial communities and impacting host biology. Accumulating data support the notion that phageomes can serve as potential biomarkers of pathological states and disease predictors. Although the mechanisms underlying these interactions remain to be fully deciphered, the potential for phage-based diagnostics, therapeutics, and microbiome interventions is immense. Understanding the phageome, with its complex taxonomy and ecological dynamics, will be key to unlocking new frontiers in host–microbe–virus interactions.

## EDITOR'S NOTE

**Ed. Note:** The author of this commentary served as the handling editor for the original article.
